# Long-Term Outcomes of Surgical Decompression in Treating Calcifying Odontogenic Cyst Associated With Compound Odontoma: A 9-Year Follow-Up Case Report and Literature Review

**DOI:** 10.1155/crid/2844784

**Published:** 2025-01-06

**Authors:** Álvaro Alarcón, Rodrigo Barison, Benjamín Martínez, Sergio González

**Affiliations:** ^1^Oral Pathology, Faculty of Medicine and Health Sciences, Universidad Mayor, Santiago, Chile; ^2^Private Practice, Chile

**Keywords:** calcifying odontogenic cyst, case report, compound odontoma, decompression

## Abstract

A calcifying odontogenic cyst (COC) is a cystic lesion originating from odontogenic epithelium, exhibiting ameloblastomatous features and containing focal accumulations of ghost cells. The standard treatment for COC typically involves enucleation followed by surgical curettage. However, if the cyst is large or closely associated with anatomical structures, decompression may be considered as a preliminary step before enucleation. A 12-year-old male patient presented with swelling in the anterior mandibular region. Radiological assessment revealed an extensive radiolucent area crossing the mandibular midline, accompanied by radiopaque areas within the lesion. The diagnosis of COC associated with compound odontoma was confirmed. The treatment plan involved decompression, followed by enucleation. After over 9 years of follow-up, the patient showed satisfactory and effective outcomes, with no signs of recurrence. This therapeutic approach minimizes the morbidity and cost associated with extensive and invasive reconstructive surgeries.

**Summary**

• A calcifying odontogenic cyst (COC) is a lesion of the head and neck that is treated with simple enucleation in the majority of cases.
• However, clinicians should be aware that some cases can be treated conservatively, considering decompression before enucleation to maintain the patient's oral structures and minimize comorbidities.

## 1. Introduction

The COC is a cystic lesion that originates from odontogenic epithelium, with ameloblastomatous characteristics and containing focal accumulations of ghost cells [1, 2]. In 1962, Gorlin et al. first described this entity [3]. Also known as “Gorlin's cyst,” it represents a lesion with varied clinical, radiographic, and histopathological features that have generated discussion and, therefore, numerous revisions regarding its classification within head and neck lesions throughout history.

Since 2017 and even in the recent update by the World Health Organization (WHO) in 2022 [4], it has been classified as one of the developmental odontogenic cysts based on cystic behavior and specific clinical characteristics. It explains that it originates from remnants of the dental lamina and also constitutes part of the spectrum of lesions composed of ghost cells [1, 4–7].

Clinically, it is asymptomatic and exhibits slow growth [6]; thus, the patient may present persistence of primary teeth [5]. When the cyst grows to large sizes, it can cause facial asymmetry and swelling covered by healthy mucosa [2].

Radiographically, the lesion can be observed as a radiolucent entity of variable size, well-defined, usually unilocular, and may have scalloped borders [7]. Additionally, in one-third to half of cases, it may contain small irregular calcified bodies with a density similar to a tooth [8].

Histopathologically, this lesion consists of an epithelium composed of low cuboidal or columnar cells, with features resembling squamous epithelium or ameloblastic-like epithelium. Masses of ghost cells can be observed. These elements can be seen within the epithelial thickness, in the fibrous capsule, or inside of adjacent connective tissue. Ghost cells are large and composed of a sizeable eosinophilic cytoplasm with an empty space in their center, indicating the lack of a nucleus [3, 5, 7–9].

The treatment of choice and the most commonly used involves enucleation, followed by curettage of the surgical site in a single session. Decompression of the lesion, known as two-stage management, can also be performed before enucleation. This approach offers several advantages that have been highlighted in recent studies, such as reducing the size of the cyst, making subsequent enucleation less invasive, and lowering the risk of damage to adjacent structures. Additionally, it reduces surgical morbidity, enhancing patient outcomes, and reducing postoperative complications [10, 11].

In this study, we report a case of an extensive COC associated with a compound odontoma, diagnosed and treated through decompression prior to enucleation at the diagnostic and emergency service of the School of Dentistry at Mayor University in Santiago, Chile. The objective is to highlight the effectiveness of conservative surgical techniques, particularly decompression, as a viable treatment option. The positive outcomes observed 9 years postintervention underscore the long-term benefits of incorporating decompression in the management of such lesions.

## 2. Case Report

A 12-year-old male patient was presented to the diagnostic and emergency service at the School of Dentistry at Mayor University, Santiago, Chile, in 2014. The patient reported a painless swelling in the mandible that had persisted for 6 months. The patient's medical history was noncontributory, with no significant medical conditions, known allergies, or current medication use. Informed consent for the publication of this case was obtained.

**Figure 1:**
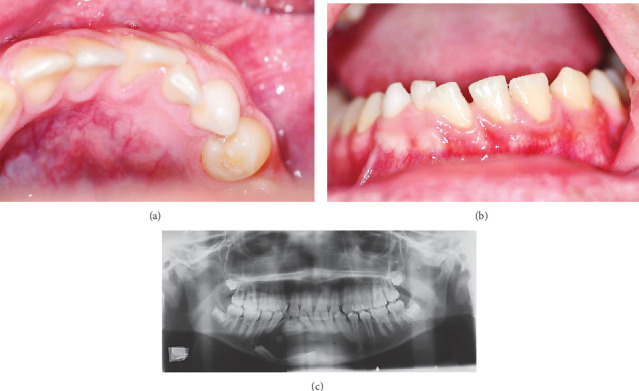
(a) Occlusal view revealing vestibular and lingual volume enlargement with intact covering mucosa. (b) Persistence of deciduous teeth and displacement of Teeth 4.1, 3.1, 3.2, and 3.3. (c) Panoramic radiograph: a well-defined radiolucent extending from Teeth 3.4 to 4.5, with calcified elements within the lesion. Tooth 4.3 displaced caudally.

Upon intraoral examination, firm swelling in the mandible was observed, extending towards both the buccal and lingual aspects (Figure 1(a)). There was persistence of primary teeth in the lower anterior region (8.2 and 8.3), absence of a permanent tooth (4.3), and noticeable displacement of the teeth in the affected mandibular quadrant, without any signs of mobility. The mucosa covering the area appeared normal (Figure 1(b)).

Radiographic assessment through a panoramic x-ray (Figure 1(c)) taken in 2014 revealed a well-demarcated radiolucent area with corticated borders, extending from the periapical region of Teeth 3.4 to 4.5 and from the alveolar ridge to the mandibular base. Cone beam computed tomography (CBCT) revealed irregular hyperdense areas within a broader hypodense region. The lesion caused the displacement of the roots of the right premolars distally and the lower central and lateral incisors towards the left side. Furthermore, Tooth 4.3 was displaced caudally and projected towards the mandibular base. Additionally, images depicted a well-defined hypodense area with corticated borders, causing expansion and thinning of both mandibular cortices without signs of perforation. Hyperdense areas near the buccal bone were suggestive of calcifications at the lesion's margin.

The surgical approach involved an intraoral vestibular incision for an incisional biopsy. During the procedure, calcifications and a portion of the membrane surrounding the lesion were observed. A white-yellowish mass, containing structures resembling denticles, was excised. Following excision, a decompression cannula was placed, creating communication from the lesion to the oral mucosal surface, where it was sutured in place. The surgical site was irrigated with a 0.12% chlorhexidine solution, followed by a follow-up 24 h later and again at 7 days. These follow-ups continued until a definitive diagnosis was established to determine the appropriate treatment plan.

**Figure 2:**
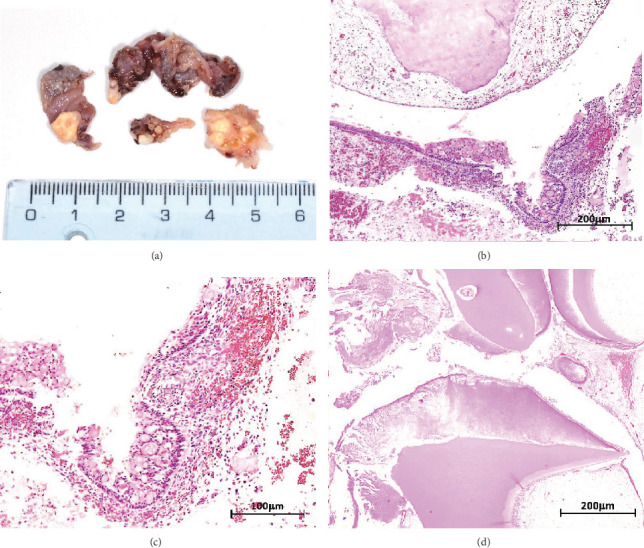
(a) Fragments of irregular brown-blackish fragments of tissue. A conglomerate of calcification denticle-like structures is evident. (b) (HE–4x) Connective tissue, partially covered by a lining epithelium. Calcified area within connective tissue, compatible with dentinoid, and interstitial hemorrhage areas are visible. (c) (HE–10x) The epithelium is composed of cells with cuboidal morphology and squamous features and is reminiscent of odontogenic epithelium. The basal layer displays palisading and hyperchromatism. Epithelial surface exhibits clusters of ghost cells. (d) (HE–4x) Denticle-like structures made by enamel matrix, dentin, and dental papilla tissue, surrounded by loose connective tissue.

Macroscopic examination revealed four dark-brown fragments, irregularly shaped, with a firm consistency. Additionally, whitish masses with denticle-like shapes were observed (Figure 2(a)). Histopathological analysis showed a structure comprising a fibrous capsule and connective tissue, partially lined by stratified epithelium. Within the connective tissue, deposits of calcified eosinophilic material consistent with dentinoid were present (Figure 2(b)). The cystic epithelium consisted of cuboidal cells with squamous characteristics, showing areas resembling the stellate reticulum. Basal cells exhibited hyperchromatism and slight palisading. Clusters of ghost cells, characterized by their eosinophilic cytoplasm and central empty spaces, were also noted extending throughout the thickness of the epithelium (Figure 2(c)). Adjacent to these findings, calcified structures with denticle-like morphology were found, consisting of an enamel matrix, dentin, and a central zone resembling dental papilla and pulp tissue (Figure 2(d)). These histopathological findings, including the presence of ghost cells, an ameloblastomatous epithelium, dentinoid material, and denticle-like structures, led to a diagnosis of a COC associated with a compound odontoma.

Once the diagnosis was confirmed, a follow-up plan was established. This involved introducing 5–10 mL of the solution through the decompression cannula with a syringe twice a day, allowing the liquid to exit after washing the cavity. Monthly follow-ups were conducted to reposition the cannula, ensure its patency, and clean the area. Panoramic radiographs were periodically taken to assess changes, such as lesion size reduction and new bone formation. The first radiograph was obtained 1 month after surgery, with subsequent images taken at 3-month, 6-month, and annual intervals.

**Figure 3:**
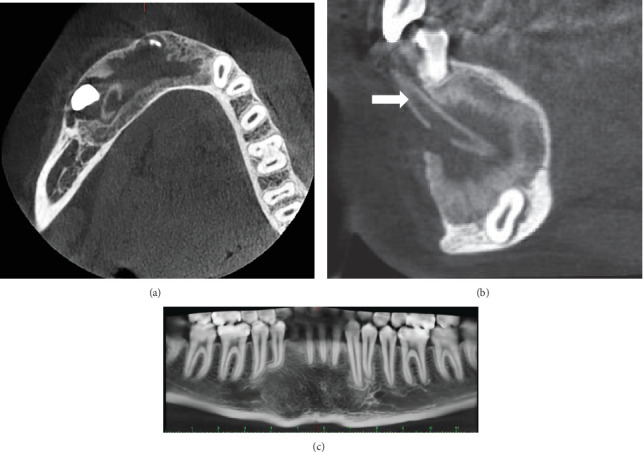
(a) CBCT axial section: 1-year follow-up (2015). Formation of trabecular bone at the lesion periphery and significant reduction in size are observed. (b) CBCT sagittal section: 1-year follow-up (2015). Peripheral new bone formation with reduced vestibule-lingual bulging is evident. The arrow indicates the cannula system. (c) CBCT panoramic view: 9-year postsurgical intervention (2023). Formation of bone tissue without alteration. Maintained anatomical structures and no signs of recurrence.

Once the cystic lesion had sufficiently reduced and adjacent structures were confirmed to be intact, a CBCT scan was conducted, followed by surgical enucleation and the extraction of the impacted tooth. This decision was made after 1 year of follow-up, based on positive radiographic indicators (Figures 3(a) and 3(b)). The patient continued with regular follow-up examinations, and the most recent CBCT, taken in 2023, showed no signs of recurrence (Figure 3(c)).

## 3. Discussion

Since the original publication by Gorlin et al. [3], there has been ongoing debate regarding the nature and classification of this entity. In the first two editions of the WHO's “Classification of Head and Neck Tumours” [12, 13], the COC was categorized as a benign odontogenic tumor. However, it was explicitly described as a non-neoplastic cystic lesion. In the second edition, the WHO classification [13] further refined this definition, classifying it solely as a cystic lesion, characterized as a histopathological variant of lesions featuring ghost cells.

In the 2005 edition of the WHO classification, this entity was renamed as the “calcifying odontogenic cystic tumor” and redefined as a benign cystic neoplasm [14]. However, during that period, researchers such as Ledesma-Montes et al. [15]; Hong, Ellis, and Hartman [16]; and Toida et al. [17] argued that most lesions with ghost cells were primarily cystic in nature, sometimes associated with an odontoma, and exhibited benign behavior with low recurrence rates. These authors suggested that the entity should be categorized within developmental cysts. In the 2017 fourth edition of the WHO classification, the original terminology was restored, reclassifying the lesion once again as a “calcifying odontogenic cyst” and identifying it as a developmental odontogenic cyst [1]. This classification was maintained in the 2022 fifth edition, which also introduced molecular aspects from a pathogenic perspective [4].

COC originates from remnants of the dental lamina, also known as Serres' remnants, which become entrapped in the maxillary bones during the tooth formation process [7]. Mutations in the CTNNB1 gene, which encodes for *β*-catenins, have been implicated in the pathogenesis of the lesion. These mutations, particularly those affecting phosphorylation sites on the protein, may sustain active signaling pathways that promote cystic growth. Moreover, such mutations might contribute to the formation of ghost cells in these types of lesions [18]. This alteration has been identified in protein accumulations within the cytoplasm of the epithelial cells lining the cystic cavity and in the nuclei of the cells surrounding the ghost cells [18–20].

It is an extremely rare lesion, accounting for less than 1% of all odontogenic cysts [21]. While COCs are recognized within the pediatric population, specific prevalence rates remain underreported in the literature. The available studies suggest that these cysts are part of a broader category of odontogenic lesions commonly encountered in children [22], but further research is needed to establish precise prevalence figures and enhance understanding of their clinical implications in the pediatric demographic.

The COC does not exhibit a predilection for the mandible or maxilla, nor does it show a gender preference. The average age of presentation is 33 years, with cases reported across a wide age range, from 5 to 92 years. The most common site of occurrence is the incisor and canine region, accounting for approximately 65% of all cases [2, 5, 6, 8]. COCs are often associated with odontomas, particularly in the second decade of life, with studies reporting this association in 24%–47% of cases [23]. Additionally, these cysts are frequently linked to retained teeth, occurring in about 10%–32% of cases [5, 7, 8, 24].

COC exhibits distinct histopathological characteristics that are crucial for accurate diagnosis. One of the hallmark features is the presence of ghost cells. These cells are identified by their eosinophilic cytoplasm and a central empty space, signifying the absence of a nucleus, which gives rise to their name. This phenomenon has been interpreted as an altered form of keratinization [25]. However, it is important to note that ghost cells are not exclusive to COC; they are also found in other lesions, including ameloblastoma, ameloblastic fibroma, ameloblastic fibro-odontoma, and odontoma. Another significant histopathological feature of COC is the presence of dentinoid deposits, typically located adjacent to the epithelial lining. These deposits are thought to result from the inductive influence of odontogenic epithelium on nearby mesenchymal tissue [26]. Immunohistochemical analysis has revealed the presence of amelogenin within ghost cells, along with the expression of CK19, Bcl-2, and Ki-67 in the cyst epithelium. These findings support the odontogenic origin of COC and highlight its proliferative nature [25].

Radiographically, COC often presents similarly to other odontogenic cystic lesions but has a strong association with odontogenic tumors. In approximately 24% of cases, COC may show structures with a density resembling that of a tooth, potentially indicating the presence of an odontoma. Additionally, a retained tooth is observed in 10%–32% of cases. When calcified densities or tooth-like structures are identified with the lesion, it is important to consider differential diagnoses. These may include adenomatoid odontogenic tumor, calcifying epithelial odontogenic tumor, ossifying fibroma, fibro-odontoma, or odontoma [8, 27].

The treatment for COC varies and can involve different surgical procedures, ranging from complete enucleation of the lesion in a single session to two-stage treatments that begin the promotion of decompression of the cyst prior to enucleation. In two-stage treatments, decompression can be achieved through marsupialization, traditional decompression, or active decompression and distraction sugosteogenesis (ADDS). Marsupialization and decompression both involve creating an opening from the cyst's lumen to the exterior, but they differ in technique. Decompression uses a sutured tube (cannula) attached to the mucosa to maintain the opening, while marsupialization involves suturing the mucosa directly to the cystic wall, creating communication between the lesion and the exterior without the use of a tube. ADDS, on the other hand, actively employs negative intracystic pressure to push the cyst or its parts outward, thereby stimulating bone regeneration (sugosteogenesis). All three techniques share the underlying principle of reducing intracystic pressure, whether passively or actively [10]. Regardless of the surgical approach, recurrence of COC is rare, occurring in less than 5% of cases [2, 6, 7].

The therapeutic approach used in this case was decompression with a cannula, chosen based on the clinical, radiographic, and histopathological characteristics of the lesion. While nearly 70% of COCs are treated with enucleation without prior decompression, conservative management is particularly important for large lesions [28]. Creating long-term communication between the lesion and the oral cavity using devices like cannulas addresses key factors in cystic growth: it directly reduces hydrostatic pressure within the cavity, facilitates the free drainage of its contents, and significantly decreases the size of the lesion [6]. Moreover, this approach offers better three-dimensional control over surrounding structures, encourages bone tissue formation and growth, and reduces facial inflammation [2, 6, 28, 29].

Decompression preserves the inferior alveolar nerve, maintains mandibular contour, and supports normal facial development, particularly in pediatric or young patients who are still growing. It also helps prevent mandibular fractures and reduces the risk of recurrence, contributing to an overall better quality of life [29]. This treatment approach offers a more favorable prognosis not only for the cyst itself but also for the surrounding tissues [28].

However, decompression has several limitations. It often involves prolonged treatment duration [30], which can take months or even years to achieve the desired outcomes. The rate of cyst reduction is also unpredictable [30, 31], varying based on factors such as sex, initial cyst volume, and location. While some studies suggest that larger cysts may have a higher reduction rate, this is not guaranteed, making outcomes difficult to predict [31, 32]. Additionally, this technique may need additional surgery [31, 33]. Even after successful decompression, a secondary surgery is often required to completely remove the cystic lesion. Patient compliance is another challenge [34], as decompression requires strict hygiene maintenance and regular follow-up appointments. Noncompliance can lead to suboptimal outcomes, including inadequate cyst reduction or complications. Furthermore, decompression is only suitable for certain types of cysts and limited to specific cases [35]. Not all COCs are amenable to decompression. Certain aggressive behaviors may require more immediate surgical intervention rather than a conservative approach, limiting the applicability of decompression as a first-line treatment [35].

## 4. Conclusion

The COC has undergone significant changes in classification over the years, reflecting ongoing debates about its nature. This entity is rare, accounting for less than 1% without a clear prevalence in the pediatric population. Treatment approaches vary, with complete enucleation being common, though decompression techniques offer a conservative alternative for larger lesions, particularly in younger patients. However, these techniques come with challenges, such as prolonged treatment duration and the potential need for additional surgical interventions. Overall, while COC generally has a favorable prognosis with low recurrence rates, its management must be tailored to the individual patient, taking into account the lesion's size, location, and the patient's compliance with treatment protocols. Continued research is essential to further refine treatment strategies and improve outcomes for patients with this rare and complex lesion.

## Data Availability

The data used in this study are included in the article.

## References

[1] Speight P. M., Takata T. (2018). New tumour entities in the 4th edition of the World Health Organization Classification of Head and Neck Tumours: odontogenic and maxillofacial bone tumours. *Virchows Archiv*.

[2] de Moraes A. T. L., Soares H. A., Viana Pinheiro J. J., Ribeiro Ribeiro A. L. (2020). Marsupialization before enucleation as a treatment strategy for a large calcifying odontogenic cyst: case report. *International Journal of Surgery Case Reports*.

[3] Gorlin R. J., Pindborg J. J., Clausen F. P., Vickers R. A. (1962). The calcifying odontogenic cyst–a possible analogue of the cutaneous calcifying epithelioma of Malherbe. An analysis of fifteen cases. *Oral Surgery, Oral Medicine, and Oral Pathology*.

[4] Soluk-Tekkesin M., Wright J. M. (2022). The World Health Organization classification of odontogenic lesions: a summary of the changes of the 2022 (5th) edition. *Turkish Journal of Pathology*.

[5] Gallana-Alvarez S., Mayorga-Jimenez F., Torres-Gómez F. J., Avellá-Vecino F. J., Salazar-Fernandez C. (2005). Calcifying odontogenic cyst associated with complex odontoma: case report and review of the literature. *Medicina Oral, Patología Oral y Cirugía Bucal*.

[6] Emam H. A., Smith J., Briody A., Jatana C. A. (2017). Tube decompression for staged treatment of a calcifying odontogenic cyst-a case report. *Journal of Oral and Maxillofacial Surgery*.

[7] El-Naggar A. K., Chan J. K. C., Takata T., Grandis J. R., Slootweg P. J. (2017). The fourth edition of the head and neck World Health Organization blue book: editors' perspectives. *Human Pathology*.

[8] Kler S., Palaskar S., Shetty V. P., Bhushan A. (2009). Intraosseous calcifying cystic odontogenic tumor. *Journal of Oral and Maxillofacial Pathology*.

[9] Woo S.-B. (2023). *Oral Pathology, A Comprehensive Atlas and Text*.

[10] Moreno-Rodríguez P., Guerrero L. M., Gómez-Delgado A., Castro-Núñez J. (2021). Active decompression and distraction sugosteogenesis for the treatment of calcifying odontogenic cyst. *Oral and Maxillofacial Surgery*.

[11] Berretta L. M., Melo G., Mello F. W., Lizio G., Rivero E. R. C. (2021). Effectiveness of marsupialisation and decompression on the reduction of cystic jaw lesions: a systematic review. *The British Journal of Oral & Maxillofacial Surgery*.

[12] Kramer I. R., Pindborg J. J., Shear M. (1992). The WHO *histological typing of odontogenic tumours*. A commentary on the second edition. *Cancer*.

[13] Pindborg J. J., Kramer I. R. H., Torloni H. (1971). *Histological Typing of Odontogenic Tumours, Jaw Cysts, and Allied Lesions*.

[14] Barnes L., Eveson J. W., Reichart P., Sidransky D. (2005). *World Health Organization Classification of Tumours: Pathology and Genetics of Tumours of the Head and Neck*.

[15] Ledesma-Montes C., Gorlin R. J., Shear M. (2008). International collaborative study on ghost cell odontogenic tumours: calcifying cystic odontogenic tumour, dentinogenic ghost cell tumour and ghost cell odontogenic carcinoma. *Journal of Oral Pathology & Medicine*.

[16] Hong S. P., Ellis G. L., Hartman K. S. (1991). Calcifying odontogenic cyst. *Oral Surgery, Oral Medicine, and Oral Pathology*.

[17] Toida M. (1998). So-called calcifying odontogenic cyst: review and discussion on the terminology and classification. *Journal of Oral Pathology & Medicine*.

[18] Yukimori A., Oikawa Y., Morita K. I. (2017). Genetic basis of calcifying cystic odontogenic tumors. *PLoS One*.

[19] Sekine S., Sato S., Takata T. (2003). Beta-catenin mutations are frequent in calcifying odontogenic cysts, but rare in ameloblastomas. *The American Journal of Pathology*.

[20] Mulvihill C., Mhaolcatha S. N., Brady P., Mckenna J., Sleeman D., Fitzgibbon J. (2020). Calcifying odontogenic cyst: a case report. *Oral Surgery*.

[21] Akshatha B. K., Manjunath G. S., Soundarya N. (2023). Calcifying odontogenic cyst associated with compound odontoma–a rare entity. *Journal of Oral and Maxillofacial Pathology*.

[22] Summersgill K. F. (2023). Pediatric oral pathology: odontogenic cysts. *Pediatric and Developmental Pathology*.

[23] Vizuete-Bolaños M. X., Salgado-Chavarria F., Ramírez-Martínez C. M., Ramos-Nieto J. J., Vazquez-Dávalos N. M. (2022). Compound odontoma associated with a calcifying odontogenic cyst. Case report and systematic review. *Journal of Stomatology, Oral and Maxillofacial Surgery*.

[24] Uchiyama Y., Akiyama H., Murakami S. (2012). Calcifying cystic odontogenic tumour: CT imaging. *The British Journal of Radiology*.

[25] Yoshida M., Kumamoto H., Ooya K., Mayanagi H. (2001). Histopathological and immunohistochemical analysis of calcifying odontogenic cysts. *Journal of Oral Pathology & Medicine*.

[26] Yeh T. H., Chen Y. C., Lee Y. P., Chiang C. P. (2022). Calcifying odontogenic cyst treated by marsupialization and subsequent total enucleation. *Journal of Dental Sciences*.

[27] Souza L. N., Souza A. C., Gomes C. C. (2007). Conservative treatment of calcifying odontogenic cyst: report of 3 cases. *Journal of Oral and Maxillofacial Surgery*.

[28] de Arruda J. A. A., Monteiro J. L. G. C., Abreu L. G. (2018). Calcifying odontogenic cyst, dentinogenic ghost cell tumor, and ghost cell odontogenic carcinoma: a systematic review. *Journal of Oral Pathology & Medicine*.

[29] Pogrel M. A. (2005). Treatment of keratocysts: the case for decompression and marsupialization. *Journal of Oral and Maxillofacial Surgery*.

[30] Kwon Y. J., Ko K. S., So B. K. (2020). Effect of decompression on jaw cystic lesions based on three-dimensional volumetric analysis. *Medicina*.

[31] Wongrattanakarn S., Trachoo V., Kaboosaya B., Charoenlarp P., Chongruangsri N. N., Promoppatum P. (2023). Factors affecting the reduction rate of odontogenic cysts after decompression based on 3-dimensional volumetric analysis. *Imaging Science in Dentistry*.

[32] Nel C., Robinson L., Roza A. L. O. C., Vargas P. A., Nortjé C. J., van Heerden W. F. (2021). Calcifying odontogenic cysts: a 20-year retrospective clinical and radiological review. *Dento Maxillo Facial Radiology*.

[33] Sheng S., Tipton N., Chang J., Meng H. W., Tribble G. D. (2023). Peripheral calcifying odontogenic cyst: a case report and comprehensive review of 60 years of literature. *Oral Health*.

[34] Samir M. C., Lamiae G., Bassima C. (2021). Calcifying odontogenic cyst of anterior maxillary: case report and review. *International Journal of Surgery Case Reports*.

[35] Strokov S., Cardot-Leccia N., Raybaud H. (2024). Cysts of the jaws and how to make their diagnoses under a microscope: a need for a better communication between clinicians and pathologists. *Journal of Oral Medicine and Oral Surgery*.

